# Comparative Study of Bulgarian Linden Honey (*Tilia* sp.)

**DOI:** 10.3390/foods14020175

**Published:** 2025-01-08

**Authors:** Anastasiya Yankova-Nikolova, Desislava Vlahova-Vangelova, Desislav Balev, Nikolay Kolev, Stefan Dragoev, Biljana Lowndes-Nikolova

**Affiliations:** 1 Department of Meat and Fish Technology, Technological Faculty, University of Food Technologies, 26 Maritza Blvd., 4002 Plovdiv, Bulgaria; d_vangelova@uft-plovdiv.bg (D.V.-V.); d_balev@uft-plovdiv.bg (D.B.); n_kolev@uft-plovdiv.bg (N.K.); s_dragoev@uft-plovdiv.bg (S.D.); 2 Bulgarian Academy of Sciences, 1040 Sofia, Bulgaria; 3 Department of Economics, Entrepreneurship and Management, Faculty of Economics, University of Food Technologies, 26 Maritza Blvd., 4002 Plovdiv, Bulgaria; biljana@mellifera.bg

**Keywords:** lime honey, HMF, diastase, sensory, melisopalynological analysis, antioxidant activity, geographical regions

## Abstract

The present study aims to evaluate and compare some of the main indicators characterizing Bulgarian linden honey depending on the geographical origin. A total of 18 samples were collected from the six regions of Bulgaria, with 3 samples from each region taken from different producers during the 2023 harvest. The physicochemical indicators: hydroxymethylfurfural content, diastase activity, pH, color, water content and electrical conductivity, as well as organoleptic and pollen characteristics, were analyzed. Antioxidant activity was also investigated by several methods: total phenolic content (TPC), phenolic compounds by the Glories method, 2,2-diphenyl-1-picrylhydrazyl (DPPH) radical activity, CUPRAC (Cupric Reducing Antioxidant Capacity), iron-reducing antioxidant capacity (FRAP) assay, and radical scavenging capacity in terms of ABTS•+, ORAC (oxygen radical antioxidant capacity). Differences were found depending on the region. All the studied honeys from the Northern Central Region contained higher *Tilia* sp. pollen. In the Southwestern Region, *Tilia* sp. pollen was not detected in any of the honey samples. The highest sensory score was awarded to linden honey from the Northern Central Region. An overall assessment ranks linden honey from the Northern Central region, the richest in linden forests, as the highest quality among the six studied regions. Another key finding was that 39% of linden honey labeled or declared as monofloral linden honey on the Bulgarian market does not meet the established criteria for monofloral honey.

## 1. Introduction

Honey is used both as a medicine and as a source of food. According to the Codex Alimentarius and EU Directive 110/2001 [1], honey is defined as a natural sweet substance produced by Apis mellifera bees from the nectar of plants or from secretions of living parts of plants or excretions of plants-sucking insects on the living parts of plants, which the bees collect, transform by combining with specific substances of their own, deposit, dehydrate, store, and leave in honeycombs to ripen and mature. Honey is a naturally sourced food product and is the only natural sweetener that humans can consume without processing, making it highly significant from an economic perspective [2].

The bee honey contains hundreds of compounds belonging to various chemical groups. In addition to the primary components, such as glucose, fructose, sucrose, water, organic acids, minerals, and amino acids, honey also contains polyphenolic compounds, enzymes, pigments, vitamins, essential oils, and other active substances [3].

Natural linden honey is a valuable source of biologically active compounds and is easily and quickly absorbed by the body [4]. Bulgaria is rich in biodiversity and favorable climatic conditions, creating an ideal environment for producing high-quality bee products. Among the most prized is linden honey, predominantly derived from the floral nectar of linden trees (*Tilia* sp.).

Natural forests of silver linden (*Tilia tomentosa*), endemic to Bulgaria, Romania, and Serbia, cover small areas within the country. These linden forests are an essential resource for collecting the medicinal “linden blossom” and serve as a vital food source for bees [5].

In the Bulgarian market, a significant part of honey is sold directly by beekeepers to consumers in packaging such as jars, buckets, or tins. However, beekeepers sometimes struggle to provide accurate information about the botanical origin and quality of their honey due to various factors. For instance, few of them analyze the pollen composition of honey, and the information they have often relies on their experience and production methods (e.g., seasonal timing and hive location). Some beekeepers may lack sufficient education or training, while others might intentionally provide misleading information to sell their products more easily.

The quality of honey is regulated by national and international standards. However, due to the previously mentioned challenges, the Bulgarian market often features honey with varying characteristics, some of which may not meet these standards. A comparative analysis of the characteristics and quality of linden honey from all regions has not been conducted in Bulgaria.

For this reason, the current study aims to evaluate and compare some of the key parameters that define Bulgarian linden honey from the 2023 harvest, focusing on its characteristics across different geographic regions.

## 2. Materials and Methods

### 2.1. Honey Samples and Study Area

**Figure 1:**
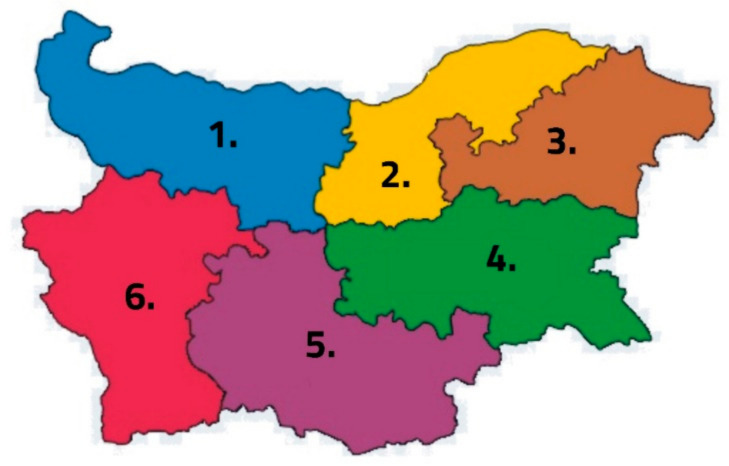
Geographical map of Bulgaria showing the six main regions.

Linden honey is the most characteristic, widespread, and preferred monofloral honey in Bulgaria. For the purposes of this study, 18 samples (3 from each region) were collected from the six official regions (districts) of Bulgaria (Figure 1). Each region provided three samples of linden honey produced by different beekeepers from the 2023 harvest. The six regions included in the study are as follows: (1) Northwestern Region; (2) North Central Region; (3) Northeastern Region; (4) Southeastern Region; (5) South Central Region; (6) Southwestern Region.

The first digit of each sample number corresponds to the geographic region in Bulgaria where the honey was collected.

Table 1 show the details about the location of the apiaries—this information is provided by the samples suppliers.

The honey samples were purchased from the Bulgarian market, directly from beekeepers or traders. All suppliers declared that their honey was either monofloral linden honey or polyfloral honey containing linden nectar.

Three jars of linden honey from the same batch were obtained from each producer, and the analyses were conducted on a composite sample created by mixing these jars. According to Bulgarian legislation, the pollen composition of monofloral nectar honey must include at least 30% pollen from the corresponding plant species for it to qualify as linden honey [6].

### 2.2. Methods

#### 2.2.1. Pollen Analysis

The pollen analysis of honey was carried out according to BDS 3050-80 [7]. This method is microscopic, based on “Methods of melissopalynology” (1978) [8].

Preparation of the honey sample: 10 g of honey was dissolved in a centrifuge tube in 20–40 cm^3^ of distilled water. The solution was centrifuged for 10–15 min at 2500–3000 min^−1^. The liquid was poured off, and a drop of the precipitate was transferred to a glass slide. After drying, the sample was fixed with a drop of pure or slightly stained with basic fuchsia alcohol.

The prepared honey sample was observed under a microscope and 150–200 pollen grains were counted, noting their species composition. The percentage of pollen grains was determined by the amount of pollen grains counted from a given plant relative to the total number of grains counted.

#### 2.2.2. Physicochemical Properties

The primary physicochemical characteristics pH, moisture content, electrical conductivity, diastase activity, and HMF content were analyzed following the Harmonized Methods of the European Honey Commission [9]:

- pH—dissolving 10 g sample in 75 mL of carbon dioxide-free water in a 250 mL beaker. Stirred with a magnetic stirrer, immersion of the pH electrodes in the solution and the pH recorded.
- Water content is determined using a refractometer. Directly after melting, if needed, and homogenization of the sample, the surface of the prism is covered evenly with the sample. After 2 min, the refractive index is ready to be recorded. The water content is the value determined from the refractive index of the honey by reference to a standard table.
- Electrical conductivity—the electrical conductivity of honey is defined as that of a 20% weight in volume solution in water at 20 °C, where the 20% refers to the honey dry matter. The result is expressed in milliSiemens per centimeter (mS.cm^−1^)/microsiemens per centimeter (µS.cm^−1^).
- Diastase activity—the unit of Diastase Activity, the Gothe unit, is defined as the amount of enzyme which will convert 0.01 g of starch to the prescribed end-point in one hour at 40 °C under the conditions of the test. The results are expressed as Gothe units (or Schade units) per gram of honey.
- HMF content—the determination of the hydroxymethylfurfural (HMF) content is based on the determination of UV absorbance of HMF at 284 nm. In order to avoid the interference of other components at this wavelength, the difference between the absorbances of a clear aqueous honey solution and the same solution after the addition of bisulfite is determined. The HMF content is calculated after subtraction of the background absorbance at 336 nm. This method is based on the original work of White.

Honey color (mmPfund) was determined using a HI-96785 Honey Color Grade portable photometer (Hanna Instruments, Bedfordshire, UK) using the Pfund honey color grading system. Instrumentally captured CIE color characteristics (L*a*b*) were determined using a CR-410 portable chromameter (Konica Minolta, Tokyo, Japan).

#### 2.2.3. Antioxidant Properties

- Total Phenolic Content (TPC)

Total phenolic content was determined by mixing 1 g of honey sample with 5 mL of 40% methanol/acidified water (*v*/*v*, pH = 2, HCl). The samples were then stirred for 15 min with a magnetic stirrer. From the extract, 0.2 mL was mixed with 2 mL of Folin–Ciocalteu reagent 1:10 and 1.8 mL of Na_2_CO_3_ 7.5% (*w*/*v*). The samples were kept in the dark for 20 min and the absorbance was measured at 750 nm using a spectrometer. Gallic acid solutions with concentrations in the range of 0–400 mg·L^−1^ were used to obtain the calibration curve. Absorbance was measured at 750 nm with UV-VIS on a Camspec Ltd., Leeds, UK spectrophotometer [2].

- Phenolic compounds by the method of Glories

The content of total phenols, phenolic acids, and flavonoids was determined by a modified method of Glories [10] at three wavelengths—280, 320, and 360 nm using a UV-VIS spectrophotometer (Camspec Ltd., Leeds, UK). Methanolic extract (1 mL) was mixed with 1 mL of 0.1% HCI in ethanol 95% *v*/*v*, 18.2 mL of 2% HCl *v*/*v* and absorbance was measured after 15 min. The results were presented as gallic acid equivalent (CAE·L^−1^) for TPC, caffeic acid equivalent (CAE·L^−1^) for phenolic acids, and quercetin equivalent (QE·L^−1^) for flavonoids [11,12].

- 2,2-Diphenyl-1-picrylhydrazyl (DPPH) radical activity

The determination of 1,1-diphenyl-2-picrylhydrazyl (DPPH) radical activity was performed by dissolving 1 g of honey in 5 mL of methanol 40% (*v*/*v*, with acidified water) and stirring for 15 min with magnetic stirring. Then, 35 µL of copper solution was mixed with 250 µL of DPPH. The absorbance was measured at 515 nm using a spectrometer (UV-VIS Camspec Ltd., Leeds, UK) [2]. The results were expressed as % DPPH using equation:

% DPPH = (*A*_0_ − *A*_1/_*A*_0_) × 100,

where *A*_0_ is the DPPH absorbance, and *A*_1_ is the absorbance of the sample.

- Radical scavenging capacity relative to ABTS•+

Antioxidant activity by the ABTS method (2,20-azinobis-(3 ethylbenzothiazoline-6-sulfonate)) was determined by the method described by Shopska et al. [11]. The absorbance was measured at 734 nm against a methanol blank and the results were expressed as µmol TE·g^−1^.

- Iron-reducing antioxidant capacity (FRAP) assay.

The ferric reducing antioxidant potential (FRAP) is based on the reduction of Fe^3+^ to Fe^2+^ in an acidic medium and the formation of a colored complex of ferro-tripyridyltriazines. The FRAP assay was performed according to Benzie and Strain [13] with some modifications from Dinkova et al. [14]. The FRAP reagent was prepared by mixing 2.5 mL of TPTZ solution (2,4,6-tris(2-pyridyl)-s-triazine) (10 mmol·L^−1^) in 40 mmol·L^−1^ HCl, 2.5 mL aq. FeCl_3_ solution (20 mmol·L^−1^), and 25 mL acetate buffer (0.3 mol·L^−1^, pH 3.6). A total of 250 μL of test methanol solution and 2250 μL of FRAP reagent were mixed in the UV-macro cuvette. After 4 min in the dark at room temperature, the absorbance was measured at 593 nm against a blank in which the extract was replaced with pure methanol. The results were expressed in µmol TE.g^−^^1^ (Trolox Eq) [12].

- CUPRAC (CUPric reducing antioxidant capacity)

The CUPRAC assay was performed as described by Shopska et al. [11]. The methanolic extracts (0.5 mL) were mixed with 1 mL of 0.01 M CuCl_2_ 2H_2_O, 1 mL of ammonium acetate buffer (pH 7), 1 mL of 7.5 × 10^−^^3^ M ethanolic solution of neocuproine, and 0.6 mL of distilled water. After 30 min, the absorbance at 450 nm was measured. The results are expressed as µmol TE·g^−1^.

- Oxygen Radical Absorbance Capacity (ORAC)

ORAC was measured according to the method of Ou, Hampsch-Woodill, and Prior [15] with some modifications. The method measures antioxidant scavenging activity against peroxyl radical induced by 2,20-azobis-(2-amidino-propane) dihydrochloride (AAPH) at 37 °C. Fluorescein (FL) was used as a fluorescent probe. The loss of FL fluorescence is an indication of the extent of damage from its reaction with the peroxyl radical. The protective effect of an antioxidant is measured by evaluating the area under the fluorescence decay curve (AUC) compared to a blank sample in which no antioxidant is present. Solutions of AAPH, fluorescein, and Trolox were prepared in phosphate buffer (75 mmol·L^−1^, pH 7.4). Samples are also diluted in a phosphate buffer. The reaction mixture (total volume 200 mL) contains FL (170 μL, final concentration 5.36 × 10^−^^8^ mol·L^−1^), AAPH—(20 μL, final concentration 51.5 mmol·L^−1^) and sample—10 mL. The FL solution and sample were incubated at 37 °C for 20 min and AAPH (dissolved in 37 °C buffer) was added. The mixture was incubated for 30 s before the initial fluorescence was measured. Then, fluorescence readings were taken at the end of each cycle after shaking. For the blank, 10 µL of phosphate buffer was used instead of the sample. Antioxidant activity is expressed in Trolox equivalents. Trolox solutions (6.25, 12.5, 25, 50, and 100 µmol·L^−1^) were used to determine the standard curve. One ORAC unit is defined as the protective area provided by a 1 µmol·L^−1^ solution of Trolox. The final ORAC values were calculated using a regression equation between Trolox concentration and the net area under the curve. The results are expressed as micromol Trolox equivalents per gram of dry extract [16].

#### 2.2.4. Sensory Analysis

Sensory analysis is a scientific method for evaluating the organoleptic properties of foods and plays an important role in establishing the authenticity of products. The research method includes the basic human senses involved in the reception and evaluation of honey as a food product. Color, aroma, taste, texture, and visual perception in addition to honey viscosity are key factors in determining honey quality and sensory acceptability [17].

The participants in the organoleptic analysis were previously trained. A honey sommelier introduced the participants to the method of conducting sensory analysis of the honey. The assessors took part in several consecutive sessions where they were trained to recognize the properties of linden honey. This procedure was carried out in accordance with ISO 8586:2023. The panel consisted of 8 evaluators between the ages of 20 and 50, 4 women and 4 men. The room was suitable for the sensory test—clean, odorless, set at room temperature, with natural light. The honey samples were placed in small transparent glass jars of 20 mL each. To refresh the receptors in the mouth between samples, the tasting panel used neutral-tasting apples and bread.

All parameters were evaluated on a numerical scale (Gonnet system) as follows:

- Visual assessment (appearance, color, purity): 0–5 points;
- Aroma (authenticity, matching the type): 0–4 points;
- Taste (authenticity, matching the type): 0–8 points;
- Texture (consistency, crystallization): 0–3 points.

All experimental samples were compared to a control sample of linden honey (linden honey from the Popovo region, Northeastern Bulgaria, harvest 2023) with the following characteristics, corresponding to a maximum score (20 points):

- Pollen analysis: *Tiliaceae* 54.1%, *Asteraceae* 26%, *Rosaceae* 12.1%, *Fabaceae* 4.4%, *Lamiaceae* 1.7%, and *Brassicaceae* 1.7%;
- Visual assessment: light to medium yellow;
- Physical characteristics: fine crystals;
- Olfactory evaluation: strong smell, chemical, and fresh;
- Taste assessment: medium sweet, chemical and minty, and long and persistent astringent aftertaste.

The same control sample was used during the training of panel for sensory properties.

The evaluation was carried out on the basis of a comparison of the sensory characteristics with those of the honey control sample. The more the studied parameter differs (does not match) with the control, the smaller the number of points it receives. Each evaluator, in addition to numerical values, also gave a description (qualities, shortcomings or other notes) where he considered it necessary. To determine the specific taste and aroma characteristics of the honey, the tasting panel used the categorization of a honey aroma “wheel” (Table 2), presented by the International Honey Commission (IHC) [18]. The sum of the individual sensory characteristics formed the overall score for each of the 18 samples.

#### 2.2.5. Statistical Analysis

- All assays were performed in triplicate. One-way ANOVA (*p* = 0.05) was used for descriptive statistics.
- Principal component analysis (PCA).

Exploratory Data Analysis (EDA) can reveal insights about intrinsic data dependencies. Principal Component Analysis were employed in this study. The focus of the analysis is on grouping the honey as function of geographical location. Each of the honey characteristics is used as data feature in EDA.

## 3. Results and Discussion

### 3.1. Pollen Analysis

The pollen spectrum (Table 3) shows that 39% of the honey samples labeled as linden honey (seven samples) do not meet the legal requirement for monofloral honey (>30% linden pollen) as per Bulgarian legislation.

The highest *Tilia* sp. (linden) pollen content was found in samples 2.1, 2.2, and 2.3 from the North Central Region: 78%, 77%, and 75%, respectively. The data reveals a lack of *Tilia* pollen in all the samples from Region 6 (Southwestern region—samples 6.1, 6.2, and 6.3).

Honey from District 1 has *Tilia* pollen content similar to that of Region 2 (North Central). In sample 1.1, it is 60%, and in sample 1.2, 78%. However, no *Tilia* pollen was found in sample 1.3.

All samples from Districts 3 (Northeast), 4 (Southeast), and 5 (South Central) contain *Tilia* pollen grains (3.1–8%, 3.2–65%, 3.3–68%, 4.1–60%, 4.2–42%, 4.3–15%, 5.1–54%, 5.2–11%, 5.3–35%). However, in samples 3.1, 4.3, and 5.2, the percentage of linden pollen is below 30%, meaning that these cannot be labeled as monofloral linden honey under Bulgarian legislation.

**Figure 2:**
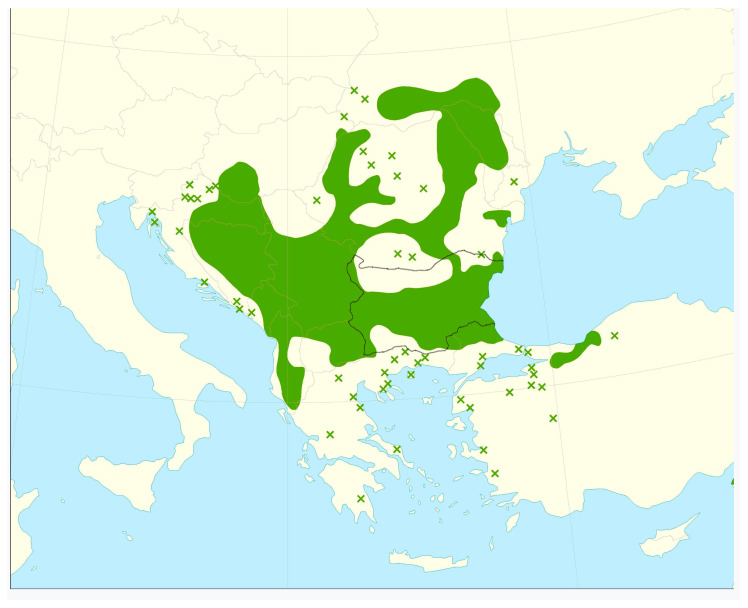
Distribution map of Tilia tomentosa (silver lime) of the Balkan Peninsula (Bulgaria borders are black marked)—native continuous range in green, native isolated population marked with green ×, according to Caudullo et al. (2017) [19].

The results obtained were synchronized with the location of linden forests as described by Tzonev (2003) [5]. In Bulgaria, silver linden forests (*Tilia tomentosa*) are primarily distributed in the Danubian Plain and Northeastern Bulgaria (Ludogorie), with a more limited presence in the Eastern Fore–Balkan region. These forests were found at elevations ranging from 50 to 60 m and 800 to 1000 m. They are typically located in hilly and pre-mountainous areas on loess or limestone substrates, predominantly on slopes with northern and eastern exposure, with inclinations ranging from 5 to 45°. Less frequently, in the Ludogorie region, they can also be found on ridges and relatively flat terrains [5]. The map (Figure 2) show Bulgarian linden forests (*Tilia tomentosa*, silver lime) distribution in 2016 [19].

### 3.2. Physicochemical Properties

The acidity and water content in honey are parameters that influence the development of yeasts and molds in the honey [3]. Most honeys are acidic, with a pH lower than 7 [20].

In the studied samples, the pH ranged from 3.88 (sample 6.2) to 5.51 (sample 2.2), (Table 4). The obtained results are typical for linden honey and confirm previous studies on linden honey from Romania, Poland, Serbia, and Slovakia, where the pH is in the range 3.6–5.4 [21]. Lower pH inhibits the growth of microorganisms and extends the honey’s shelf life [22].

In the present study, the moisture content ranged from 15.4% (sample 5.3) to 19.4% (sample 5.2) and did not exceed 20% (according to the Bulgarian regulation on honey intended for human consumption, effective from 28 January 2023, adopted with Government Decision No. 3 from 6 January 2023), confirming the quality and stability of the honey during storage. As the refractive index is higher, the lower is the moisture content in honey. Low moisture content prevents fermentation and increases the shelf life and storage time of honey [23]. According to the results, the region of origin (areas 1 to 6) does not affect the moisture content of the honey (*p* < 0.05).

HMF (hydroxymethylfurfural) is one of the most important criteria for monitoring honey quality. Improper handling, related to increased temperatures during honey processing and improper storage [24], causes HMF levels to rise above the allowable limit of 40 mg/kg according to Bulgarian legislation. In fresh honey, HMF levels are naturally low, but its concentration increases with storage time and prolonged heating. Thermal processing at high temperatures for extended periods destroys vitamins and bio-nutrients. At the same time, the HMF content increases, and diastase activity decreases [25]. HMF is also used to check for adulteration of honey with glucose syrup [26]. According to the criteria and standards for honey [27], the allowable HMF limit is 40 mg/kg. In all the tested samples, the detected HMF was below the maximum allowable value, ranging from 1.38 ± 0.53 mg/kg (sample 1.3) to 22.12 ± 4.00 mg/kg (sample 4.3). The obtained results for HMF confirm the quality of the honey, indicating no thermal processing or poor storage conditions [28].

According to the honey quality requirements of the EU Council Directive, diastase activity must be above 8 Schade units, expressed as diastase number (DN). DN in the Schade scale, corresponding to the Goethe units, is defined as 1 g of starch per 100 g of honey, hydrolyzed for 1 h at a temperature of 40 °C [29]. The results show that eight out of the tested samples (44.4%) have a diastase number below 8 Schade units. In Region 4, (Southeastern) DN in all three tested samples was found below the required minimum value.

The results for electrical conductivity (Table 4) show that the lowest values for linden honeys were found in sample 4.2 (494 ± 1.41 µS·cm^−1^), while the highest was in sample 3.3 (858 ± 7.07 μS·cm^−1^). This parameter correlates with the content of electrolytes in honey, including various metal and inorganic compounds. In a similar study by Albu et al. [21], the highest average value of 506 μS·cm^−1^ was found in Romanian linden honey.

Compared to most nectar honeys (up to 0.8 mS·cm^−1^), linden honey is an exception and does not have a defined standard for electrical conductivity values (Bulgarian regulations and Directive 2001/110/EC). Comparing the six regions, high conductivity were found in all samples from Region 2 (627 ± 4.24; 670 ± 2.83; 834 ± 2.83 μS·cm^−1^). The highest was electrical conductivity in sample 3.3 (858 ± 7.07 μS·cm^−1^). However, according to Lazarieva et al. [4], the electrical conductivity in high-quality linden honey should not exceed 0.63 mS·cm^−1^ (or 630 μS·cm^−1^, n.a.).

In all tested samples (including non-linden ones), the electrical conductivity was found in normal range according to Directive 110/2001 of the EU and Bulgarian regulations for honey intended for human consumption. Since electrical conductivity is used to verify the botanical origin and purity of honey, Vîjan et al. [27] stated that for polyfloral honey, this parameter ranges between 500 and 800 μS·cm^−1^, with an upper limit of 689 μS·cm^−1^ for polyfloral honey from Bulgaria.

According to the instrumentally measured color characteristics, the highest value of color lightness (L) was found in samples 5.1 (78.39 ± 1.20), followed by 1.2 (75.12), 4.3 (74.88), and 5.2 (74.40). The instrumental CIE Lab* color characteristics include lightness (L*), red-green coordinate (a*, −a*), and yellow-blue coordinate (b*, −b*). Using the L* value, the lightness or darkness of the sample can be determined (100 is white and 0 is black) [28,30].

The data obtained for the color components (Table 4) show that some of the a* values are positive (darker honeys on the Pfund scale), meaning that red color predominates, while those with negative values show a green tint (lighter ones). We can conclude that groups with higher L* values have a negative a* value, indicating that lighter colors have a greenish color. The highest *a*∗ value is in sample 3.3, 3.73 ± 0.84 (with the strongest red tint), which is also one of the darkest honeys. The strongest green tint was found in sample 1.3 with (a*—4.25 ± 0.40).

The obtained b* values for all the tested samples were positive, meaning that all samples have a yellow hue, with no blue tint observed.

Linden honey’s color varies between white and light amber, while polyfloral samples tend to have darker shades [21]. According to the Pfund scale, the color ranges from white to light amber. The lowest value of 25 mmPfund (white) on the Pfund scale was obtained for sample 1.1, while the highest value of 69 mmPfund was found for sample 2.3 (darkest, linden). Sample 6.2 with a value of 17 mmPfund is excluded from the conclusions, as the pollen analysis (Table 3) shows that it is not linden honey, but predominantly contains pollen from *Rosaceae* and *Fabaceae* families.

**Figure 3:**
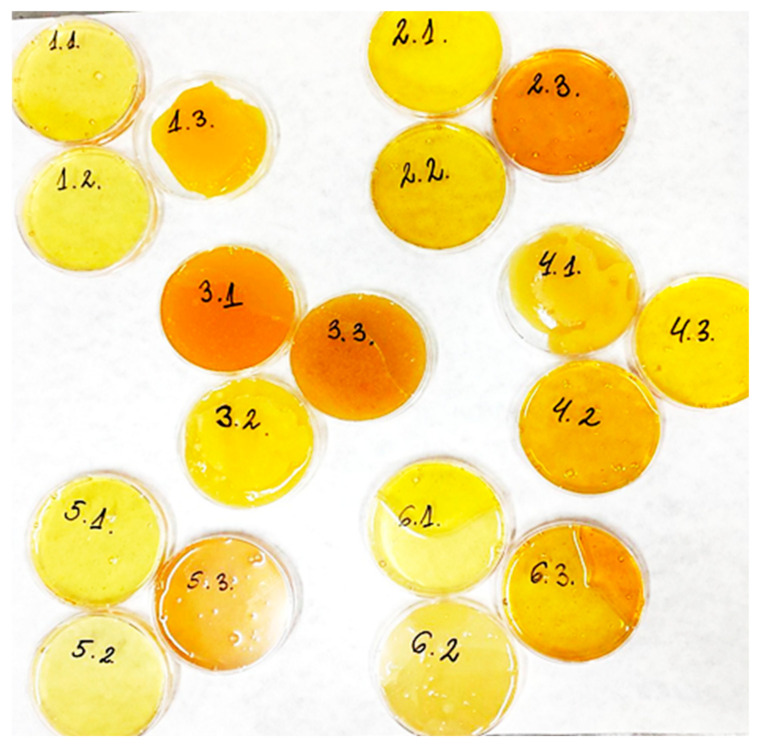
Visualization of honey samples (honey is placed in transparent Petri dishes with transparent lids, on white paper).

Visually (Figure 3), some of the samples show a deviation from the normal, light color typical for linden honey. It is known that the intensity of the color correlates positively with certain organic compounds in honey (total phenols and flavonoids), as well as with their induced antioxidant activity. Furthermore, darker-colored honey is likely richer in minerals compared to lighter honey.

The significantly darker and atypical color for linden honey was observed in samples 1.3, 2.3, 3.1, 3.3, 4.2, and 6.3. These findings are confirmed by the pollen analysis (Table 3), which shows that honey from samples 1.3 (Northwest region) and 6.3 (Southwest) does not contain linden pollen, and sample 3.1 (Northeast region) contains only 8% linden pollen. In the other darker-colored samples (2.3, 3.3, and 4.2), the linden pollen content exceeds 30%, and the color deviations are likely due to other impurities.

### 3.3. Antioxidant Activity

The functional properties of honey are determined by the antioxidants contained in the pollen. The antioxidant effect of honey is due to the presence of phenolic acids, flavonoids, ascorbic acid, carotenoids, catalase, peroxidase, etc. [30].

According to the data in Table 5, the maximum value of TPC was found in sample 6.1, 5203.68 ± 6.08 mg GA·kg^−1^, but the honey is not linden. The pollen assay (Table 3) shows that 39% of the pollens in sample 6.1 were represented by Lotus. Higher values of TPC were established in honey from the Northeast region (3.3) with a TPC value of 3376.91 ± 5.02, respectively, mg GA·kg^−1^ and South Central Region (5.3, 4386.37 ± 3.35 mg GA·kg^−1^). Compared to Bulgarian honey, TPC and TFC in linden honey from China and Russia ranged from 17.57 to 31.95 mg GAE·100 g^−1^ and 0.81 to 1.77 mg RE·100 g^−1^ [31]. It is well established that the content of phenolic compounds in honey is strongly influenced by its botanical and geographical origin. On the other hand, the composition of bioactive components depends significantly on the floral source and region where the honey is produced. These variations directly impact antioxidant activity [32,33] and explain the observed differences in total phenolic content (TPC) and total flavonoid content (TFC).

Total phenolics (mg GAE·kg^−1^) in linden honey from the six studied Bulgarian regions ranged from 196.33 ± 3.32 to 1833.00 ± 3.84. For comparison, honey from Kurdistan, harvest 2022, has a much lower total phenolic content (mgGAE·100 g^−1^) and is in the range of 30.8 ± 3.9 to 79.6 ± 6.6 [34].

The lowest content of phenolic acids (110.73 ± 2.16; 187.97 ± 2.23; 289.59 ± 2.13) was found in honey from Districts 6—Southwest, but the pollen composition of all three samples indicates that it is not linden (Table 3). A similarly low phenolic acid content was found in sample 1.3 (182.28 ± 2.70) from the Northwest region, which is also not linden honey. The content of phenolic acids in honey from Districts 2—North Central was ten times higher (1243.25 ± 3.66; 1325.37 ± 2.52; 1387.97), which correlates with the highest established content of linden pollen (77%; 78%; 75%). Three more samples are distinguished by a high content of phenolic acids: sample 1.2 from Districts Northwest (1128.62 ± 2.58), sample 3.3 from Northeast region (1231.87 ± 2.79), and sample 5.3 South Central Region (1193.66 ± 2.74). According to the pollen analysis (Table 3) the content of linden pollen in samples 1.2, 3.3, and 5.3 honeys was 78%, 68%, and 35%, respectively. However, samples 3.3 and 5.3 seriously deviate in color according to the sensory requirements for linden honey, showing a dark and uncharacteristic color (Figure 3).

Phenolic compounds (PC) in the honey samples determined by the Glories method were significantly lower compared to TPC by the FC method (Table 5). A similar difference in the study of phenolic content by the two methods was reported in studies by Aleixandre-Tudo et al. [35] and Shopska et al. [11]. One possible reason may be the main disadvantage of the FC method is that the reaction used to quantify the presence of phenolic compounds is based on SET [35]; therefore, any substance possessing electron donor properties, even if it is not a phenolic compound (organic acids, proteins etc.), will be quantified as such.

In sample 2.3 from the North Central Region the values of total phenols and phenolic acids content, as well as antioxidant activity determined by the ABTS and FRAP methods were found highest. DPPH value of sample 2.3 was high, but not the highest compared to all studied samples. Both the DPPH and ABTS methods are based on reduction by single electron transfer (SET) or hydrogen atom transfer (HAT). It is accepted that in the DPPH method, the transfer is slower due to the difficult accessibility of the active site of the DPPH molecule. One possible reason for the low established value of antioxidant activity measured by DPPH is the fact that different amounts and types of phenolic compounds potentially block access to DPPH• radicals [33].

According to Shuai Zou et al., the antioxidant capacity of linden honey ranges from 4.51 to 9.38 mg Vitamin C·100 g^−1^ in DPPH assays, 10.89 to 34.83 mg Vitamin C·100 g^−1^ in ABTS assays and 30.88 to 51.73 mg Vitamin C·100 g−1 in FRAP assays in the samples from China and Russia, observing the same tendency for DPPH values to be lower than ABTS and FRAP [31].

In a study on different Hungarian honeys by Farkas et al., the IC _50 values_ from the TEAC assay (ABTS•+) were the highest for linden honey (130.34 ± 12.86 IC _50_ mg mL^−1^), which means that its antioxidant power is significantly lower than that of other types of honey, supported by its TRC value. However, in the ORAC analysis, the value for linden honey is surprisingly high (44.33 ± 5.38 μmol TE·g^−1^), despite being from the group of light-colored honeys. [36]

In the present study, the highest values for ORAC were found in sample 4.3, 1423.9 ± 5.7 µmol TE·100 g^−1^ (which is not linden honey), sample 4.1, 1267.1 ± 22.4 µmol TE·100 g^−1^, and sample 2.2, 1177.1 ± 13.6 µmol TE·100 g^−1^, followed by 4.2 and 2.3. (Southeast and North Central Regions resp.). The obtained ORAC values for honeys from Regions 2 and 4 are in sync with the data for phenolic content.

The highest CUPRAC values were recorded in sample 2.2 (7273.68 ± 3.37 µmol TE·L^−1^), followed by samples 4.1 (5794.19 ± 4.25 µmol TE·L^−1^) and 4.2 (4640.16 ± 2.98 µmol TE·L^−1^). Once again samples from Region 2 (North Central) and region 4 (Southeast) have the highest antioxidant activity determined by CUPRAC assay, highest content of phenols and phenolic acids and strong correlation between CUPRAC phenols and phenolic acids.

Dumbravă et al. [37] reported a CUPRAC value of 0.375 µmol Trolox·g^−1^ for Romanian lime honey from Timiș County, which means that antioxidant activity measured by CUPRAC in Bulgarian linden honey from North Central and South East regions is higher than Romanian [37]. In a study by Aktaş et al., all honey samples from Turkish chestnut honey have a high CUPRAC value (11.0–97.1 mmol Trolox·100 g^−1^) [38], and Kaygusuz et al. [39] found a CUPRAC value of 23.8 ± 1.5 to 17.18 ± 1.52 µmol Trolox·g^−1^ in a chestnut honey sample originating from Trabzon [38], which have higher values, but the honey were not linden.

### 3.4. Sensory Analysis

Linden honey (family *Tiliaceae*) has specific characteristics with a strong aroma that is fresh and vegetal with some chemical notes. It is medium sweet, with moderate acidity and a persistent astringent aftertaste. Linden honey forms a compact mass of fine crystals, light yellow in color, sometimes with greenish tints.

The sensory characteristics of linden honey samples were evaluated against a control sample with pre-determined features, representing the highest sensory quality for linden honey and receiving the maximum score of 20 points.

The highest sensory score, compared to all other honey samples (Table 6), was awarded to sample 1.2 (Northwestern Region) with a total score of 18.1, closest to the maximum of 20 points. The results correspond to the pollen analysis data, showing the highest linden pollen content (78% *Tilia*).

Following sample 1.2, the next highest-ranked samples were sample 5.1 with 16.8 points (54% *Tilia* pollen) from the South Central Region, and sample 3.2 with 16.5 ± 2.52 points (65% *Tilia* pollen) from the Northeastern Region.

The three samples from Districts 6 (6.1–7.6, 6.2–2.0, and 6.3–4.7) received the lowest sensory results because no linden taste and aroma were detected by the test panel. Pollen analyses (Table 3) confirmed that there were other pollens than lime. Sample 6.1 had predominant pollens from *Lotus* (39%) and *Trifolium* (31%). In sample 6.2, 41% of pollens were represented by the *Rosaceae* family and 39% by *Fabaceae*. Sample 6.3 contained 59% of pollens from *Fabaceae*.

Sample 2.3, despite having a very high percentage of linden pollen (75%), scored only ten points due to the presence of a “foreign, non-specific taste and aroma” and a “very dark color”. Similarly, samples 2.1 (78%) and 2.2 (77%), also rich in linden pollen, did not rank among the top-scoring samples.

On the other hand, samples 1.3 and 4.3, which do not meet the linden pollen threshold for monofloral honey, received relatively high scores (15.3 and 14.5 points, respectively). These higher scores were attributed to their “light yellow color, small fine crystals” and “yellow color, fine crystals”.

Sample 3.1 (Northeastern Region) received a total of 8.8 points, with its taste described as “caramel, atypical for linden”. This result aligns with the pollen analysis, which confirmed that it is not linden honey, containing 33% *Foeniculum* and 27% *Helianthus annuus* pollen.

**Figure 4:**
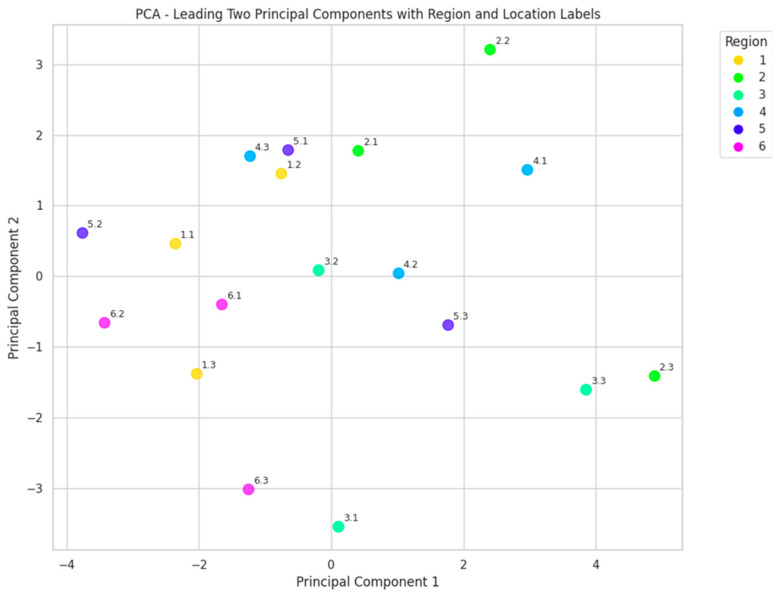
Location distribution based on the leading components of PCA.

PCA was carried out to discover any statistical dependencies among samples within each region. The goal was to verify that samples collected from the same region form groups due to their similarities in properties and characteristics. Our hypothesis was that geographical proximity will cause similarity in quality but this was not confirmed. PCA was applied using all honey parameters and the clusters formed by region is depicted in Figure 4. It can be verified that all region clusters intersect and even some clusters include other clusters. The distribution of locations represented by the leading components does not reveal any clear clustering. This analysis concludes that honey properties are diverse across the country and honey similarities cannot be formed based on geographical principle.

## 4. Conclusions

Bulgarian linden honey from the Northern Central Region, from the harvest 2023 has excellent organoleptic characteristics, and according to the results for the content of polyphenols, phenolic acids, and antioxidant properties, it ranks among the highest quality linden honeys. This region is one of the richest in linden forests.

Another key finding was that 39% of honey labeled or declared as monofloral linden honey on the Bulgarian market does not meet the established criteria for monofloral honey. This is particularly evident in regions where lime forests are either absent or scarce.

The results obtained from this research represent an excellent basis that can be used to develop a methodology for identifying the characteristics of Bulgarian monofloral linden honey. The data show the need for urgent actions to strengthen the control and prevent the market of honey that does not correspond to the botanical origin.

## Figures and Tables

**Table 1:** Location of apiaries.

District	Sample	Town/Village	GPS Coordinates of the Region	Bee Type	Municipality	Region
Northwest	1.1	Mramoren village	43.298096°, 23.678137°	*Apis mellifera rodopica*	Vratza	Vratza
	1.2	Gorno Pestene village	43.277092°, 23.710482°	*Apis mellifera rodopica*	Vratza	Vratza
	1.3	Koynare village	43.355699°, 24.143577°	*Apis mellifera rodopica*	Cherven bryag	Pleven
North Central	2.1	Yuper village	43.916667°, 26.4°	*Apis mellifera rodopica*	Kubrat	Razgrad
	2.2	Dulovo village	43.822946°, 27.141233°	*Apis mellifera rodopica*	Dulovo	Silistra
	2.3	The town of Silistra	44.109238°, 27.265381°	*Apis mellifera rodopica*	Silistra	Silistra
Northeast	3.1	Avren village	43.110168°, 27.667995°	*Apis mellifera rodopica*	Avren	Varna
	3.2	Prilep village	42.849899°, 26.929908°	*Apis mellifera rodopica*	Dobrich	Dobrich
	3.3	Donchevo village	43.516667°, 27.766667°	*Apis mellifera rodopica*	Dobrich	Dobrich
Southeast	4.1	Srednogorovo village	42.531195°, 25.330981°	*Apis mellifera rodopica*	Kazanlyk	Stara Zagora
	4.2	The town of Aytos	42.698961°, 27.248972°	*Apis mellifera rodopica*	Aytos	Sliven
	4.3	Omarchevo village	42.45791°, 26.144843°	*Apis mellifera rodopica*	Nova Zagora	Sliven
South Central	5.1	The town of Kardzhali	41.644721°, 25.374966°	*Apis mellifera rodopica*	Kardzhali	Kardzhali
	5.2	The town of Simeonovrgad	42.03236°, 25.834458°	*Apis mellifera rodopica*	Simeonovgrad	Haskovo
	5.3	The town of Kalofer	42.611157°, 24.974847°	*Apis mellifera rodopica*	Karlovo	Plovdiv
Southwest	6.1	The town of Petrich	41.398129°, 23.206857°	*Apis mellifera rodopica*	Petrich	Blagoevgrad
	6.2	The town of Petrich	41.398129°, 23.206857°	*Apis mellifera rodopica*	Petrich	Blagoevgrad
	6.3	Vaksevo village	42.162079°, 22.857866°	*Apis mellifera rodopica*	Nevestino	Kyustendil

**Table 2:** Honey aroma “wheel”.

Basic Category	Sub-Category	Individual Aroma
Vegetal	green	raw bean, crumpled leave, vegetation, after the rain
	dry	pale malt, straw, tea, dry hay
Woody	dry	leafy wood, dust, walnut, hazelnut
	resinous	cedar, pine resin, propolis
	spicy	clove, nutmeg, coffee
Chemical	petrochemical	styrene, paint, solvent
	medicine	household soap, vitamin B1
Fresh	refreshing	mint, eucalyptus, aniseed
	citrus fruit	lemon, orange, grapefruit
Floral, fresh fruit	floral	orange blossom, violet, rose, hyacinth
	fruit	pear, apple, red fruit, blackcurrant, coconut, apricot, exotic fruit
Warm	burned	molasses, burned sugar
	of cooked fruit	dates, plums, figs, raisins, candied fruits
	caramelized	toffee, caramel, brown sugar
	subtle	fresh butter, vanilla, beeswax, almond paste
Spoiled	pungent	piquant cheese, vinegar
	animal	cheese, perspiration, cowshed, cat’s urine
	moldy	damp floorcloth, humus, stuffy
	sulfur	globe artichoke, cabbage

**Table 3:** Melisopalynological analysis—pollen characteristics.

Sample	*Tilia*	Fabaceae	*Helianthus annuus*	*Paliurus spina christi*	Apiaceae/Umbelliferae	Cruciferae/*Brassica*	*Coriandrum*	Asteraceae	*Foeniculum*	Rhamnaceae	Lamiaceae	Rosaceae	*Trifolium*	*Amorpha*	*Vicki*	Ericaceae/*Vaccinium*	*Lotus*	Liliaceae
1.1	60%	15%	4%	2%	2%													
1.2	78%	10%	2%		3%	1%												
1.3		12%	62%			2%	10%	3%										
2.1	78%	2%	10%				1%											
2.2	77%	2%			1%	1%		12%										
2.3	75%	7%	13%		2%													
3.1	8%	10%	27%			2%			33%	2%	1%							
3.2	65%	4%	20%			2%												
3.3	68%	5%	7%		5%						2%							
4.1	60%	15%	3%		1%													
4.2	42%	10%	26%			2%					2%							
4.3	15%	27%	29%	8%	2%	4%					1%	1%						
5.1	54%	27%	2%					1%			1%							
5.2	11%				9%	3%		1%				3%	32%	9%	3%			
5.3	35%	40%				4%				2%	1%	1%				4%		
6.1				16%									31%		3%		39%	
6.2		39%			2%	8%						41%						1%
6.3		59%			5%	10%					8%							

**Table 4:** Physicochemical properties of Bulgarian linden honey.

Sample	pH	HMF, mg/kg	Diastase Activity, Goethe Units	Water Content, %	Electrical Conductivity, μS·cm^−1^	mm Pfund	The Pfund Honey Color Grading System	L*	a*	b*
1.1	4.44 ^f^	3.20 ± 1,13 ^e^	5.84 ^g^	17.0 ^d^	348 ± 1.41 ^m^	25 ^gf^	white	68.70 ± 1.40 ^cd^	−1.78 ± 0.60 ^i^	34.49 ± 1.54 ^e^
1.2	4.93 ^b^	6.03 ± 1.49 ^d^	8.08 ^d^	17.8 ^c^	671 ± 2.12 ^d^	28 ^f^	white	75.12 ± 1.35 ^b^	−3.11 ± 0.12 ^j^	31.27 ± 2.07 ^e^
1.3	4.00 ^h^	1.38 ± 0.53 ^f^	8.48 ^d^	18.1 ^c^	283 ± 2.83 ^o^	41 ^d^	extra white amber	64.22 ± 0.60 ^d^	1.29 ± 0.42 ^ef^	56.62 ± 2.46 ^a^
2.1	4.79 ^cd^	4.79 ± 1.64 ^d^	10.20 ^c^	18.9 ^ab^	627 ± 4.24 ^f^	38 ^e^	extra white amber	71.14 ± 1.64 ^c^	−4.25 ± 0.40 ^k^	47.64 ± 1.88 ^b^
2.2	5.51 ^a^	n.d	5.52 ^g^	16.2 ^e^	670 ± 2.83 ^d^	39 ^de^	extra white amber	67.01 ± 1.06 ^d^	−0.98 ± 0.57 ^g^	42.32 ± 1.01 ^d^
2.3	4.77 ^cd^	5.92 ± 0.96 ^d^	13,20 ^a^	16.2 ^e^	834 ± 2.83 ^b^	69 ^a^	light amber	59.93 ± 2.05 ^e^	6.87 ± 1.20 ^c^	45.49 ± 1.26 ^c^
3.1	4.10 ^g^	16.52 ± 2.09 ^a^	11.56 ^b^	17.0 ^d^	479 ± 0.71 ^k^	57 ^c^	light amber	55.86 ± 1.21 ^f^	9.45 ± 0.62 ^b^	45.22 ± 1.83 ^c^
3.2	4.42 ^f^	7.94 ± 1.39 ^d^	8.24 ^d^	17.2 ^d^	545 ± 4.24 ^i^	39 ^de^	extra white amber	64.50 ± 1.95 ^d^	−0.45 ± 0.55 ^g^	55.32 ± 2.74 ^a^
3.3	4.78 ^cd^	n.d	6.84 ^f^	18.2 ^c^	858 ± 7.07 ^a^	61 ^bc^	light amber	51.70 ± 0.67 ^g^	13.73 ± 0.84 ^a^	37.70 ± 2.07 ^e^
4.1	4.97 ^a b^	n.d	4.92 ^h^	16.9 ^d^	566 ± 4.95 ^h^	43 ^d^	extra white amber	59.93 ± 0.54 ^e^	4.57 ± 0.59 ^d^	45.25 ± 0.37 ^c^
4.2	4.41 ^f^	8.60 ± 3.82 ^d^	7.28 ^e^	16.6 ^de^	494 ± 1.41 ^j^	58 ^c^	light amber	65.58 ± 2.55 ^d^	1.65 ± 1.76 ^ip^	56.96 ± 1.22 ^a^
4.3	4.50 ^e^	22.12 ± 4.00 ^a^	3.24 ^i^	16.0 ^ef^	475 ± 1.41 ^l^	44 ^d^	extra white amber	74.88 ± 1.35 ^b^	−4.81 ± 0.52 ^k^	51.87 ± 2.58 ^b^
5.1	4.82 ^c^	11.27 ± 2.16 ^b^	8.56 ^d^	17.4 ^cd^	577 ± 0.71 ^g^	32 ^f^	white	78.39 ± 1.20 ^a^	−3.26 ± 0.04 ^j^	25.95 ± 1.63 ^f^
5.2	4.85 ^c^	18.48 ± 3.45 ^a^	5.16 ^g^	19.4 ^a^	307 ± 0.71 ^n^	23 ^g^	white	74.40 ± 1.34 ^b^	−1.63 ± 0.05 ^i^	21.87 ± 1.64 ^g^
5.3	4.44 ^f^	n.d	9.80 ^c^	15.4 ^g^	647 ± 4.24 ^e^	42 ^d^	extra white amber	66.90 ± 0.51 ^d^	1.42 ± 0.62 ^ip^	41.05 ± 1.43 ^d^
6.1	4.91 ^bc^	6.54 ± 1.15 ^d^	10.08 ^c^	18.0 ^c^	721 ± 2.83 ^c^	36 ^ef^	extra white amber	70.32 ± 1.96 ^c^	−1.65 ± 0.42 ^i^	33.95 ± 2.78 ^e^
6.2	3.88 ^i^	11.90 ± 0.89 ^c^	6.44 ^f^	17.8 ^c^	550 ± 0.71 ^i^	17 ^h^	extra white	70.08 ± 0.51 ^c^	−1.36 ± 0.16 ^h^	25.88 ± 0.54 ^f^
6.3	4.00 ^h^	14.35 ± 1.49 ^b^	10.00 ^c^	16.8 ^e^	309 ± 2.12 ^n^	43 ^d^	extra white amber	66.45 ± 1.23 ^d^	2.54 ± 0.56 ^ip^	36.35 ± 0.90 ^e^

Values are presented as mean ± standard deviation. Values followed by different letters (a–p) in the same column are significantly different (*p* < 0.05).

**Table 5:** Antioxidant activity of the honey.

		Glories Method	Glories Method	Glories Method					
Sample	TPC (mg GA·kg−1)	Total Phenols (mg GAE·L−1)	Phenolic Acids (mg CAE·L−1)	Flavonoids (mg QE·L−1)	DPPH (µmol·L−1)	ABTS (µmol TE·L−1)	FRAP (µmol·L−1)	CUPRAC (µmol TE·L−1)	ORAC (µmol TE·100−1)
1.1	3456.28 ± 7.01 ^m^	751.33 ± 2.78 ^k^	583.09 ± 1.64 ^j^	NF	962.37 ± 6.21 ^k^	1090.37 ± 7.33 ^q^	1776.75 ± 7.30 ^n^	899.90 ± 4.32 ^o^	475.3 ± 7.4 ^h^
1.2	3682.22 ± 6.49 ^j^	1458.00 ± 3.07 ^g^	1128.62 ± 2.58 ^g^	NF	578.20 ± 1.07 ^p^	1881.33 ± 4.10 ^h^	2671.84 ± 4.86 ^k^	1280.99 ± 3.74 ^m^	436.3 ± 4.6 ^hj^
1.3	3781.63 ± 6.88 ^i^	328.00 ± 3.76 ^q^	182.28 ± 2.70 ^q^	NF	903.71 ± 6.48 ^l^	1233.20 ± 3.37 ^o^	2618.96 ± 4.85 ^l^	1380.24 ± 2.93 ^l^	595.2 ± 21.5 ^g^
2.1	3376.91 ± 5.02 ^n^	1688.00 ± 3.29 ^c^	1325.37 ± 2.52 ^b^	NF	1070.38 ± 3.26 ^j^	1815.07 ± 4.28 ^j^	2898.83 ± 5.91 ^i^	1462.14 ± 4.92 ^j^	913.2 ± 1.2 ^e^
2.2	4028.40 ± 4.64 ^f^	1648.00 ± 4.53 ^d^	1243.25 ± 3.66 ^d^	NF	542.52 ± 2.28 ^q^	2067.49 ± 4.01 ^g^	2614.29 ± 3.15 ^l^	7273.68 ± 3.37 ^a^	1177.1 ± 13.6 ^c^
2.3	4554.54 ± 7.61 c	1833.00 ± 3.84 ^a^	1387.97 ± 2.88 ^a^	NF	2064.52 ± 5.05 ^c^	4766.60 ± 26.80 ^a^	5220.40 ± 2.13 ^a^	2204.77 ± 3.52 ^d^	1019.2 ± 6.3 ^d^
3.1	4733.72 ± 7.28 ^b^	684.67 ± 2.64 ^l^	446.50 ± 2.45 ^m^	NF	1115.35 ± 5.67 ^i^	1729.10 ± 7.03 ^k^	4218.10 ± 6.30 ^e^	2061.02 ± 4.07 ^f^	405.7 ± 15.3 ^h^
3.2	3652.75 ± 2.84 ^k^	1166.33 ± 4.29 ^i^	911.54 ± 3.14 ^h^	NF	876.34 ± 4.74 ^m^	1629.60 ± 4.86 ^m^	2744.29 ± 6.29 ^j^	1509.80 ± 4.56 ^i^	799.4 ± 8.6 ^e^
3.3	5191.89 ± 6.50 ^a^	1619.67 ± 4.71 ^e^	1231.87 ± 2.79 ^e^	NF	1608.50 ± 7.71 ^d^	2535.19 ± 4.40 ^d^	4486.86 ± 6.31 ^d^	2261.73 ± 4.82 ^d^	742.3 ± 6.9 ^f^
4.1	4267.31 ± 7.26 ^e^	1738.00 ± 3.79 ^b^	1313.983.21 ^c^	NF	1358.75 ± 7.49 ^f^	2742.44 ± 5.89 ^b^	4132.50 ± 6.18 ^f^	5794.19 ± 4.25 ^b^	1267.1 ± 22.4 ^b^
4.2	4254.34 ± 6.68 ^e^	781.33 ± 3.38 ^j^	538.37 ± 2.55 ^k^	NF	1534.70 ± 2.55 ^e^	2243.79 ± 4.81 ^e^	4796.52 ± 5.14 ^c^	4640.16 ± 2.98 ^c^	1041.4 ± 16.7 ^d^
4.3	3785.96 ± 6.54 ^j^	668.00 ± 3.60 ^m^	496.10 ± 2.74 ^l^	NF	2074.29 ± 7.30 ^c^	1656.65 ± 4.04 ^l^	2935.64 ± 8.54 ^h^	1516.23 ± 4.45 ^i^	1423.9 ± 5.7 ^a^
5.1	3886.16 ± 7.05 ^g^	1248.00 ± 1.90 ^h^	918.05 ± 2.14 ^i^	NF	691.10 ± 5.65 ^o^	1871.24 ± 5.54 ^i^	5101.50 ± 4.62 ^b^	1539.62 ± 4.09 ^h^	794.3 ± 63.0 ^e^
5.2	3798.14 ± 5.70 ^h^	628.00 ± 10.25 ^n^	307.48 ± 2.48 ^n^	NF	1335.78 ± 7.84 ^g^	1182.31 ± 3.35 ^p^	3598.19 ± 6.59 ^g^	895.29 ± 4.46 ^o^	448.4 ± 35.1 ^h^
5.3	4386.37 ± 3.35 ^d^	1511.33 ± 3.73 ^f^	1193.66 ± 2.74 ^f^	NF	2582.60 ± 4.81 ^b^	2609.24 ± 3.63 ^c^	1486.37 ± 4.61 ^p^	2121.91 ± 4.85 ^e^	617.8 ± 22.8 ^g^
6.1	3780.06 ± 6.79 ^i^	466.33 ± 3.09 ^o^	289.59 ± 2.13 ^o^	NF	2630.99 ± 4.72 ^a^	1596.81 ± 4.99 ^n^	1987.96 ± 4.84 ^m^	1420.52 ± 5.32 ^k^	435.4 ± 0.7 ^hj^
6.2	3513.26 ± 7.93 ^l^	196.33 ± 3.32 ^r^	110.73 ± 2.16 ^r^	NF	775.66 ± 9.25 ^n^	959.69 ± 4.12 ^r^	1726.50 ± 8.67 ^o^	1156.04 ± 4.07 ^n^	412.6 ± 9.5 ^hk^
6.3	5203.68 ± 6.08 ^a^	433.00 ± 3.53 ^p^	187.97 ± 2.23 ^p^	NF	1203.32 ± 3.66 ^h^	2220.18 ± 4.05 ^f^	4481.89 ± 7.57 ^d^	1910.65 ± 5.37 ^g^	455.7 ± 4.7 ^Hi^

Values are presented as the mean ± standard deviation. (a–r) Values followed by different letters in the same column are significantly different (*p* < 0.05).

**Table 6:** Sensory evaluation/organoleptic analysis of the honey.

Sample	I. Visual Evaluation—Appearance, Color, Cleanliness (0–5 Points)	II. Aroma (0–4 Points)	III. Taste (0–8 Points)	IV. Texture (0–3 Points)	Overall Rating	Notes
1.1	4.0 ± 0.63	3.3 ± 0.82	5.71.37 ±	2.8 ± 1.33	15.8 ± 0.98	Light white/beige color, large crystals, fresh, not so strong linden taste, very sweet
1.2	4.4 ± 1.19	3.9 ± 0.35	7.1 ± 1.13	2.8 ± 0.46	18.1 ± 2.64	Light yellow color, medium and fine crystal, herbal freshness, intense taste of linden, light pleasant aroma, typical astringent aftertaste
1.3	3.9 ± 1.36	3.3 ± 0.89	5.9 ± 1.46	2.3 ± 0.71	15.3 ± 3.58	Light yellow color, small, fine crystals, very smooth structure, fruity taste and aroma, does not meet the characteristics of the linden type; short aftertaste
2.1	4.0 ± 1.00	3.3 ± 1.15	5.7 ± 2.52	2.3 ± 1.15	15.3 ± 5.69	Intense yellow color, small and round crystals, perfume aroma and fruity taste, atypical for linden, very sweet and slightly sour taste
2.2	4.5 ± 0.58	3.5 ± 1.00	4.8 ± 2.50	3.0 ± 0.00	15.8 ± 3.20	Light white/beige color, medium size crystals, weak aroma, fresh herbal, quite astringent taste.
2.3	2.3 ± 0.58	2.0 ± 0.00	3.3 ± 2.89	2.3 ± 1.15	10.0 ± 4.36	Fine crystals, atypical aroma and taste, bitter taste
3.1	3.0 ± 1.63	2.7 ± 1.53	3.7 ± 3.06	1.3 ± 0.58	8.8 ± 6.55	Light caramel color, atypical aroma and taste of caramel, too sweet, no aftertaste
3.2	4.5 ± 0.58	3.5 ± 0.58	6.0 ± 1.63	2.5 ± 1.00	16.5 ± 2.52	Creamy, fine crystals, fresh herbal aroma of linden, hint of mint, astringent aftertaste
3.3	2.0 ± 1.41	3.0 ± 0.00	7.0 ± 0.00	2.0 ± 0.00	14.0 ± 1.41	Large, coarse crystals, typical chemical plant aroma, sweet, no aftertaste
4.1	3.8 ± 0.84	2.8 ± 1.10	6.8 ± 1.10	2.0 ± 0.71	15.4 ± 3.36	Creamy, sticky, vegetal taste with a mint note, moderately sweet with a bitter aftertaste
4.2	3.0 ± 0.00	2.0 ± 0.00	5.0 ± 0.00	3.0 ± 0.00	13.0 ± 0.00	Darker than the control, less sweet
4.3	3.3 ± 1.71	3.0 ± 1.15	6.3 ± 1.50	2.0 ± 0.00	14.5 ± 4.12	Yellow color, fine crystals, pale fruity aroma, sour taste, no aftertaste.
5.1	4.4 ± 0.55	3.4 ± 0.55	6.6 ± 0.89	2.4 ± 0.55	16.8 ± 1.64	Compact and hard lumps of crystals, floral aroma, slightly sour taste of fermentation
5.2	4.0 ± 1.00	1.7 ± 1.15	4.3 ± 0.58	1.7 ± 1.15	11.7 ± 3.79	Creamy texture, sweet aroma and sour taste, fermented
5.3	2.5 ± 2.12	3.0 ± 0.00	6.0 ± 0.00	2.0 ± 0.00	13.5 ± 2.12	Fine crystal, weaker linden taste and aroma compared to the control
6.1	2.2 ± 1.64	1.4 ± 1.67	3.2 ± 3.27	0.8 ± 0.84	7.6 ± 7.09	Liquid without crystals, chestnut aroma, very aromatic sweet beginning, long aftertaste
6.2	1.0 ± 0.00	1.0 ± 0.00	0.0 ± 0.00	0.0 ± 0.00	2.0 ± 0.00	Lightly fresh and sour, lemon-like in taste only, fruity aroma, taste and aroma not matching linden
6.3	1.3 ± 0.58	1.0 ± 1.73	1.0 ± 1.73	1.3 ± 0.58	4.7 ± 4.62	Coarse crystal, association with liquid soap and medicine, taste and aroma not corresponding to linden

## Data Availability

The original contributions presented in the study are included in the article, further inquiries can be directed to the corresponding author.
